# DDX52 knockdown inhibits the growth of prostate cancer cells by regulating c-Myc signaling

**DOI:** 10.1186/s12935-021-02128-y

**Published:** 2021-08-16

**Authors:** Wandong Yu, Hangbin Ma, Junhong Li, Jinchao Ge, Pengyu Wang, Yinghao Zhou, Jun Zhang, Guowei Shi

**Affiliations:** Department of Urology, The Fifth People’s Hospital of Shanghai, Fudan University, 801 Heqing Road, Minhang District, Shanghai, 200240 People’s Republic of China

**Keywords:** RNA helicases, DDX52, Prostate cancer, c-Myc

## Abstract

**Background:**

DDX52 is a type of DEAD/H box RNA helicase that was identified as a novel prostate cancer (PCa) genetic locus and possible causal gene in a European large-scale transcriptome-wide association study. However, the functions of DDX52 in PCa remain undetermined. The c-Myc oncogene plays a crucial role in the development of PCa, but the factors that regulate the activity of c-Myc in PCa are still unknown.

**Methods:**

We determined DDX52 protein levels in PCa tissues using immunohistochemistry (IHC). DDX52 expression and survival outcomes in other PCa cohorts were examined using bioinformatics analysis. The inhibition of DDX52 via RNA interference with shRNA was used to clarify the effects of DDX52 on PCa cell growth in vitro and in vivo. Gene set enrichment analysis and RNA sequencing were used to explore the signaling regulated by DDX52 in PCa. Western blotting and IHC were used to determine the possible DDX52 signaling mechanism in PCa.

**Results:**

DDX52 expression was upregulated in PCa tissues. Bioinformatics analysis showed that the level of DDX52 further increased in advanced PCa, with a high DDX52 level indicating a poor outcome. In vitro and in vivo experiments showed that downregulating DDX52 impeded the growth of PCa cells. High DDX52 levels contributed to activating c-Myc signaling in PCa patients and PCa cells. Furthermore, DDX52 expression was regulated by c-Myc and positively correlated with c-Myc expression in PCa.

**Conclusion:**

DDX52 was overexpressed in PCa tissues in contrast to normal prostate tissues. DDX52 knockdown repressed the growth of PCa cells in vitro and in vivo. Deleting c-Myc inhibited DDX52 expression, which affected the activation of c-Myc signaling.

**Supplementary Information:**

The online version contains supplementary material available at 10.1186/s12935-021-02128-y.

## Introduction

Prostate cancer (PCa) is the leading cause of cancer mortality among males worldwide [1]. PCa patients go through a series of defined stages, including androgen-dependent and castration-resistant stages, followed by progression to invasive and metastatic cancer [2, 3]. During the progression of PCa, the major clinical challenges are to provide an effective therapeutic method with which to treat patients with advanced cancer [4]. Many key genetic changes, including the amplification of c-Myc, fusion of TMPRSS2 and ETS family genes, amplification and/or mutation of the androgen receptor, deletion and/or mutation of PTEN and TP53, and mutation of SPOP (speckle type BTB/POZ protein) have been reported to contribute to the development and progression of PCa [3]. However, there is little known about the downstream molecular regulation of these genetic alterations in PCa. Hence, it is important to understand the underlying mechanism by which these genetic alterations give rise to progressed PCa, as this will likely promote the development of effective disease therapies.

The amplification of c-Myc is a very common genetic change that is implicated in all stages of PCa [3]. The amplification of c-Myc is higher in metastatic PCa (37%) compared to primary PCa (8%) [5, 6], indicating the critical role c-Myc expression plays in the progression of PCa. Transgenic mice expressing human c-Myc generated murine prostatic intraepithelial neoplasia followed by invasive tumors [7]. However, how c-Myc functions in PCa has been largely unexplored.

DEAD/H box RNA helicases are an important group of proteins including several family members that modulate mRNA translation in cancer cells. The inhibition of these RNA helicases is a common mechanism of anti-tumor therapeutics [8]. A transcriptome-wide association study involving 79,194 cases and 61,112 controls of European ancestry identified several novel PCa genetic loci and possible causal genes including the DEAD/H box RNA helicase DDX52 [9]. However, little is known about the function of DDX52 in the progression of PCa.

In this study, we explored the expression levels of DDX52 in PCa and normal prostate tissues. Knockdown of DDX52 affected PCa cell growth in vitro and in vivo. Furthermore, DDX52 expression was regulated by c-Myc, and c-Myc signaling was inhibited following the disruption of DDX52 in PCa cells. Overall, our results strongly suggest that an increase in DDX52 levels contributes to PCa progression driven by c-Myc.

## Materials and methods

### Cell culture

The LNCaP, 22RV1 and PC3 cell lines were generously provided by the Steam Cell Bank, Chinese Academy of Sciences (Shanghai, China). Both cell lines were cultured in RPMI 1640 medium (L210KJ; BasalMedia, Shanghai, China) supplemented with 10% fetal bovine serum (S660JJ; BasalMedia), 1% penicillin/streptomycin (15070063; Gibco, Grand Island, NY, USA), and 1% HEPES (15630080; Gibco). The cells were grown at 37℃ in a humidified incubator under 5% CO_2_.

### Plasmids and lentiviral infection

The following short hairpin RNA (shRNA) expression sequences were used: DDX52, 5′-GCCAATCCAAATGCAAGCCAT-3′; c-Myc, 5′-CCCAAGGTAGTTATCCTTAAA-3′; and control, 5′-GCTCCGTGAACGGCCACGAGT-3′. These sequences were cloned into the PLKO.1 vector (10879; Addgene, Watertown, MA, USA). The overexpressed DDX52 plasmids were cloned into the pLVX-IRES-ZsGreen1 vector (Takara Bio, Shiga, Japan) via DNA assembly (#E5520; NEB, Rowley, MA, USA).

The psPAX2 plasmid (12260; Addgene) and pCMV-VSVG plasmid (8454; Addgene) in 293 T cells were transfected into HEK293T cells with PEI 25K (23966-1; Polysciences, Warring, PA, USA) following the manufacturer’s instructions. The culture medium was collected after 48 and 72 h, and the supernatant was centrifuged at 1000 rpm for 10 min to obtain the supernatant products containing the lentivirus to transduce into the indicated cells. Puromycin (5 µg/ml) (Sigma–Aldrich, St. Louis, MO, USA) was used to isolate the stable transformants in PC3 and 22RV1 cells.

### Cell growth and colony formation assay

The Cell Counting Kit-8 was used to measure cell growth according to the manufacturer’s instructions (CK04-1000T; Dojindo Technologies Inc., Rockville, MD, USA). The 22RV1 (1500 cells/well) and PC3 cells (1500 cells/well) were plated in 96-well plates and assessed at the indicated times after seeding.

The 22RV1 (700 cells/well) and PC3 cells (700 cells/well) were also seeded in six-well plates in complete medium for 6–14 days depending on the size of the colony. Then, the cells were fixed in methanol for 10 min and stained with 0.1% crystal violet for 1 h.

### Animal experiments

Approximately 1 × 10^6^ lentivirus-infected 22RV1 cells in Matrigel (1:1 [v/v], 356234; Corning, Inc., Corning, NY, USA) were injected into 6-week-old male athymic nude mice (n = 7) under anesthesia by intraperitoneal injection of 2% Tribromoethyl alcohol (125 ~ 400 mg/kg; HY-B1372; MedChem Express, Shanghai, China). A tumor-free status was defined as a xenograft that did not touch the flank of the male nude mice. All mice were sacrificed 6 weeks later, and the xenografts were dissected and weighed. The euthanasia of mice was performed by inhaled CO_2_ for the concentration of 60% for 10 min, and then to rise to 100% for 30 min. All animal experiments were approved by the Experimental Animal Ethics Committee of the Department of Laboratory Animal Science, Fudan University.

### Western blotting and IHC

The cells were washed twice in phosphate-buffered saline and solubilized in lysis buffer. Approximately 40 µg of protein sample was separated using sodium dodecyl sulfate–polyacrylamide gel electrophoresis and transferred to a polyvinylidene fluoride membrane (Immobilon IPVH304F0; Sigma–Aldrich). The membrane was blocked in 5% bovine serum albumin in Tris-buffered saline and Tween and incubated with primary and secondary antibodies.

The tissues were fixed in 10% buffered formalin for 24 h and embedded in paraffin. The paraffin-embedded tissues were sectioned and placed on charged glass slides, followed by hematoxylin and eosin or IHC staining using an IHC staining kit (G1215-200 T; Servicebio, Wuhan, China) following the manufacturer’s instructions. IHC scores were calculated using the formula: IHC score = intensity score × percentage score. The intensity score was estimated according to staining intensity (0: negative, 1: weak, 2: moderate, and 3: strong); the percentage score was determined according to the percentage of stained cells (0: 0, 1: 1–25, 2: 26–50, 3: 51–75, and 4: 76–100%). Antibodies against the following were used: DDX52 (NBP2-33776; Novus Biological, Centennial, CO, USA; western blot [WB]: 1:2,000, IHC: 1:500), c-Myc (ab32072; Abcam Cambridge, MA, USA; WB: 1:1,000, IHC: 1:100), Ki67 (sc-15,402; Santa Cruz Biotechnology, Santa Cruz, CA, USA; IHC: 1:200), AR (sc-816; Santa Cruz Biotechnology, Santa Cruz, CA, USA; WB: 1:1000), GAPDH (sc-365,062; Santa Cruz Biotechnology, Santa Cruz, CA, USA; IHC: 1:200), and vinculin (66305–1-Ig; Proteintech, Rosemont, IL, USA; WB: 1:1,000).

### RNA sequencing and analysis

Total RNA extracted from the indicated groups of 22RV1 and PC3 cells was subjected to RNA sequencing (RNA-seq) performed by Majorbio Biopharm Technology (Shanghai, China). The sequencing reads were analyzed and mapped using the free online Majorbio Cloud Platform (www.majorbio.com) to acquire the expression profiles. The sequencing data were submitted to the Gene Expression Omnibus (GEO; GSE171797).

### Bioinformatics analysis

The expression profiles published by Yu et al. (GSE6919) [10], Grasso et al. (GSE35988) [11], and Penney et al. (GSE62872) [12] were downloaded from the GEO. Expression profiles from Abida et al. (SU2C/PCF Dream Team) [13], the Cancer Genome Atlas, and Kumar et al. (Fred Hutchinson Cancer Research Center) [6] were downloaded from cBioPortal (https://www.cbioportal.org/) [14, 15]. Gene set enrichment analysis (GSEA) [16] was performed using software provided by the Broad Institute (http://www.broadinstitute.org/gsea/index.jsp) following the website’s guidelines. We used the curated gene sets (hallmark gene sets) within MSigDB.

### Statistical analysis

All statistical analyses were performed using GraphPad Prism software (version 9; GraphPad Software Inc., La Jolla, CA, USA). The statistical analysis methods are described in the figures or figure legends. A p-value < 0.05 was considered to indicate significance.

## Results

### DDX52 is associated with the development of human PCa

**Fig. 1:**
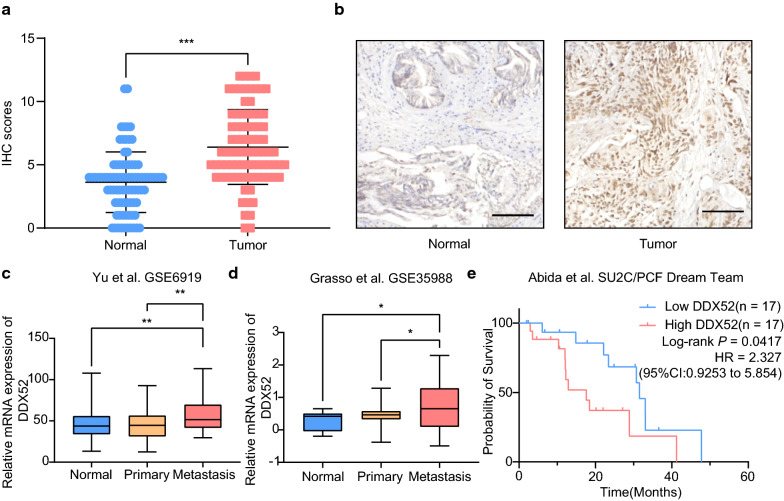
DDX52 is overexpressed in human prostate cancer (PCa) and is associated with poor outcomes. **a** DDX52 expression in normal prostate tissues and PCa tissues was examined using immunohistochemistry (IHC) (Mann–Whitney test, error bars represent standard deviations; ***p < 0.001). **b** Representative photographs of normal prostate tissues and PCa tissues (scale bar: 100 μm). **c**, **d** DDX52 expression in the indicated human prostate tissues (data derived from the Gene Expression Omnibus datasets GSE6919 and GSE35988) (*t*-test; *p < 0.05; **p < 0.01). **e** Kaplan–Meier plot revealing differences in the probability of survival between 25% higher DDX52 expression and 25% lower DDX52 expression in cohorts of advanced PCa patients

In line with a report that predicted that DDX52 expression is correlated with PCa risk [9], we determined the expression levels of DDX52 in normal (n = 85) and PCa (n = 83) tissues using IHC. The staining scores were significantly higher in tumor tissues than in normal tissues (Mann–Whitney test; p < 0.001; Fig. 1a). The staining of normal prostate tissues was weaker than that of PCa tissues (Fig. 1b). To further confirm this result, our data mining using public data sets indicated that DDX52 expression was higher in advanced PCa (metastatic tumors) than in the normal or primary counterparts (Fig. 1c, d) [10, 11]. In addition, a Kaplan–Meier plot revealed a significant association between higher DDX52 expression and shorter survival time in advanced PCa patient cohorts (Fig. 1e) [13]. Taken together, these results strongly suggest that DDX52 plays a causal role in tumorigenesis and the progression of PCa.

### DDX52 knockdown decreases PCa cell growth in vitro

**Fig. 2:**
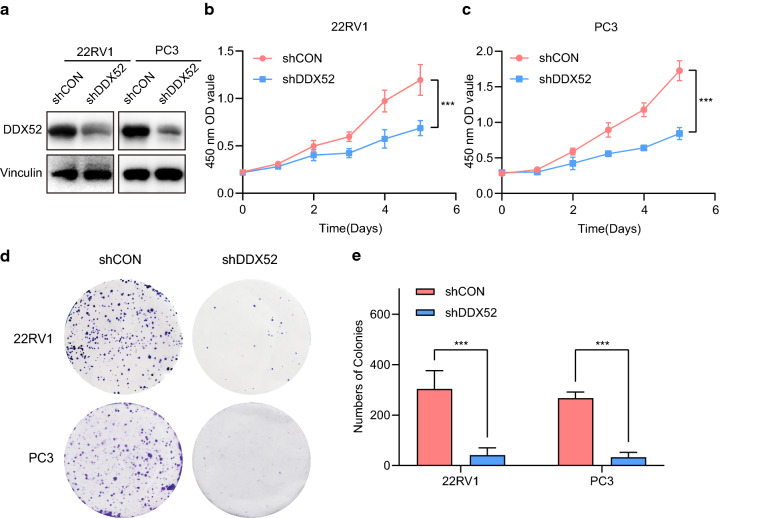
DDX52 knockdown impairs PCa cell proliferation in vitro. 22RV1 and PC3 cells were infected with lentivirus containing short hairpin RNA (shRNA) against DDX52 or the non-target control (shCON). **a** The efficiency of knockdown was verified using western blotting. Vinculin was used as the control to measure the loading quantities of the samples for western blotting. **b**, **c** 22RV1 and PC3 cells expressing shRNA targeting DDX52 or a control sequence were cultured in 96-well plates for 5 days. The growth rate in each group was measured using the Cell Counting Kit-8 assay (*t*-test, error bars represent 95% CIs; ***p < 0.001). **d** The viability of 22RV1 and PC3 cells expressing shRNA or shCON was determined using a colony formation assay. **e** Histogram showing the number of colonies in each group (*t*-test, error bars represent 95% CIs; ***p < 0.001)

To test the role DDX52 plays in PCa cell proliferation in vitro, we interrupted DDX52 using shRNA targeting DDX52. The knockdown efficiency of DDX52 was confirmed using western blotting (Fig. 2a). Deleting DDX52 in 22RV1 or PC3 cells significantly affected cell growth (Fig. 2b, c). In the cell viability experiments, we obtained a similar result, that DDX52 knockdown significantly reduced the number of cell colonies (Fig. 2d, e). These results show that inhibiting DDX52 expression suppressed PCa cell growth in vitro.

### DDX52 is required for PCa growth in vivo

**Fig. 3:**
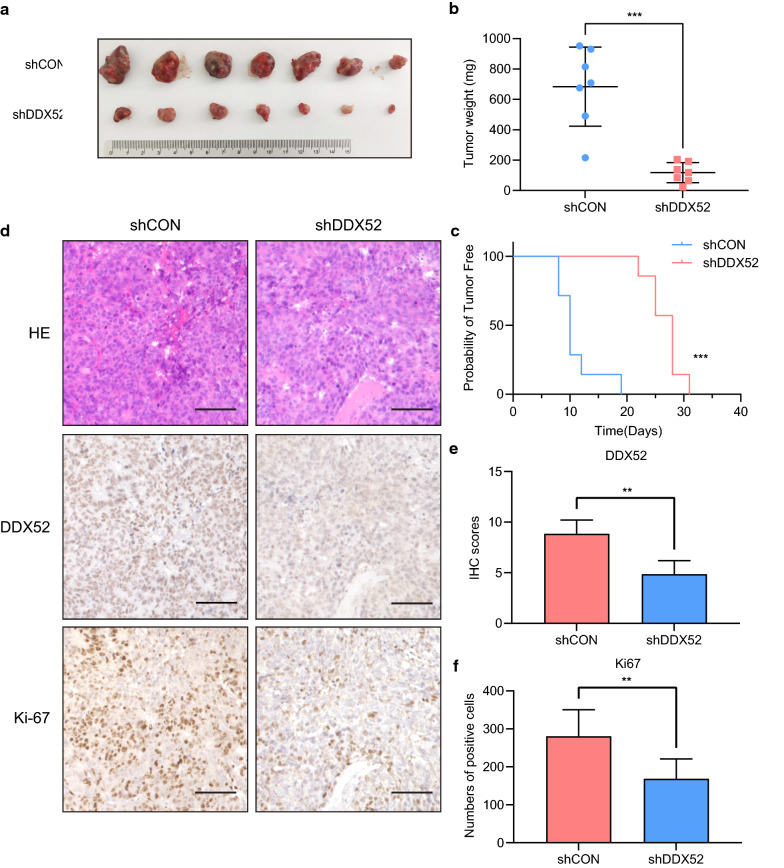
DDX52 knockdown inhibits PCa xenograft growth. **a** The mice were killed at 6 weeks after implanting 22RV1 cells, and the volumes of the xenograft tumors are shown. **b** Weights of the xenograft tumors in the control and treated groups (Mann–Whitney test, n = 7, error bars represent standard deviations; ***p < 0.001). **c** Kaplan–Meier plot showing the time of xenograft genesis after implantation (log-rank test; ***p < 0.001). **d** Examples of tumor xenografts that underwent hematoxylin and eosin (HE) staining and DDX52 and Ki67 immunohistochemical staining (scale bar: 100 μm). **e**, **f** The positive cells in random areas in each xenograft section were counted. **e** DDX52 expression was measured according to the IHC score (Mann–Whitney test, error bars represent standard deviations; **p < 0.01). **f** Ki67 expression was measured according to the number of positively stained cells (Mann–Whitney test, error bars represent standard deviations; **p < 0.01)

Next, we subcutaneously injected PCa cells with or without DDX52 downregulated into nude mice. During a 6-week follow-up, mice injected with DDX52-knockdown PCa cells displayed strongly delayed tumorigenesis (Fig. 3c). All mice were killed at the end of the study, and the xenografts were examined. Xenografts in the control group were significantly larger (Fig. 3a) and weighed more than those in the DDX52-knockdown group (Fig. 3b), demonstrating that inhibiting DDX52 significantly suppressed PCa tumorigenesis and growth in vivo. Moreover, the IHC analyses of the xenograft tissues showed that lower expression of DDX52 was correlated with reduced Ki67 expression, indicating decreased proliferation of tumor cells (Fig. 3d–f).

### DDX52 affects the activation of c-Myc signaling in PCa

**Fig. 4:**
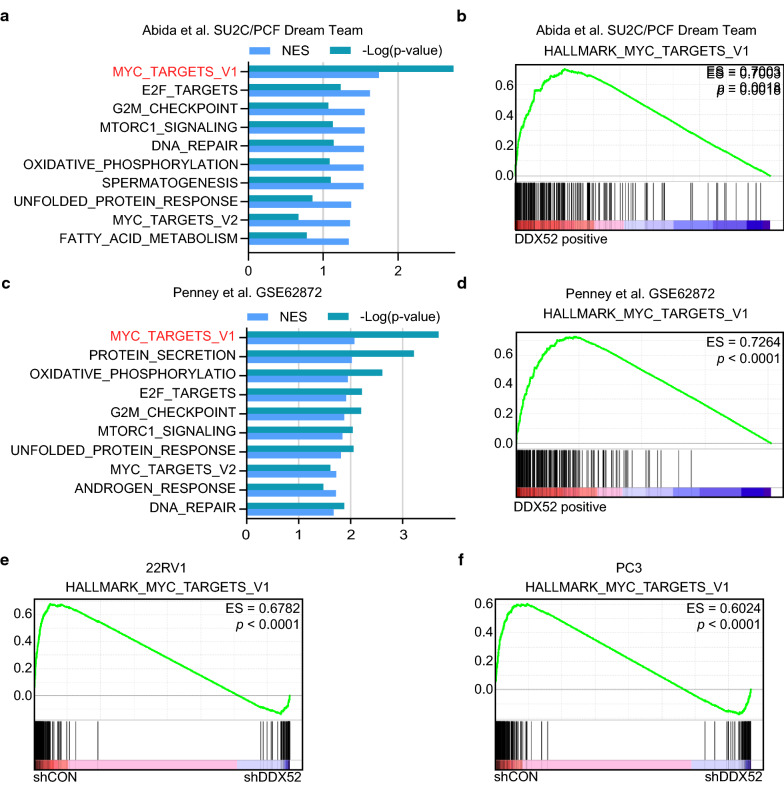
DDX52 affects the activation of c-Myc signaling in PCa. **a**, **c** Gene set enrichment analysis (GSEA) performed based on hallmark gene sets indicated that the c-Myc-upregulated gene set was the most significantly enriched in patients with high DDX52 expression in the indicated PCa cohorts. The results are shown in rank order according to the normalized enrichment score and the p-value. **b**, **d** The c-Myc-upregulated gene set was used to perform GSEA for the respective datasets based on the DDX52 transcription level. **e**, **f** GSEA of the expression profiles obtained from RNA sequencing of 22RV1 (**e**) or PC3 (**f**) cells with (shDDX52, right) or without (shCON, left) DDX52 knocked down based on the c-Myc-upregulated gene set.

To investigate the possible mechanism underlying the inhibitory effects of DDX52 in silencing PCa cell growth, we performed a hallmark pathway enrichment analysis with published PCa datasets using the GSEA method (Fig. 4a, c). The relative normalized enrichment scores and p-values, as shown in rank order, demonstrated that the c-Myc-upregulated gene set was the most significantly enriched in patients with high DDX52 expression (Fig. 4a–d). These results were confirmed in two different PCa cohorts. Furthermore, we performed RNA-seq analyses of PCa cells with or without DDX52 knockdown. Similarly, the set of c-Myc-upregulated genes was the most highly enriched in control PCa cells (Fig. 4e, f). Taken together, these data indicate that DDX52 affects the activity of oncogenic c-Myc signaling in PCa, which affects tumor progression.

### c-Myc regulates DDX52 expression in PCa

**Fig. 5:**
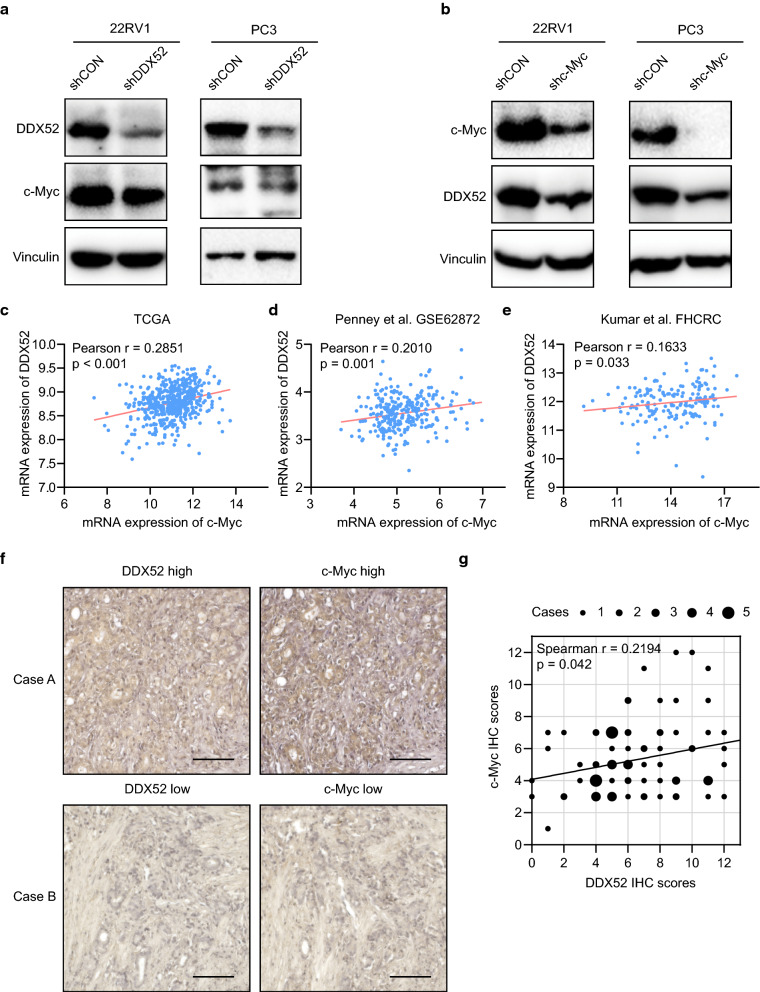
c-Myc regulates DDX52 expression in PCa. **a**, **b** 22RV1 and PC3 cells were infected with lentiviruses carrying shRNA against DDX52, c-Myc or shCON, and gene expression was determined using western blotting. **c** Published PCa datasets were analyzed to assess the correlation between mRNA expression of DDX52 and c-Myc. **f** Representative photographs of PCa tissues stained with DDX52 and c-Myc (scale bar: 100 μm). **e** Correlation between the expression levels of DDX52 and c-Myc as determined using the IHC score (n = 86)

To explore the activating mechanism of c-Myc signaling affected by DDX52 expression in PCa, we detected c-Myc expression after downregulating DDX52 in PCa cells. However, we did not discover a significant decrease in c-Myc (Fig. 5a, Additional file 1: Figure S1b). Therefore, we knocked down c-Myc in PCa cells and found that the DDX52 level decreased significantly (Fig. 5b, Additional file 1: Figure S1b). These data suggest that DDX52 is regulated by c-Myc and is required for c-Myc signaling in PCa. In addition, due to the importance about androgen receptor (AR) signaling in PCa, we also detected the expression of AR and found that both full-length and variants did not have significant change after DDX52 knockdown in PCa cells (Additional file 1: Figure S1a). To investigate whether DDX52 expression is linked to c-Myc expression in PCa, we analyzed published human PCa gene expression datasets. Strikingly, a strong positive correlation was observed between DDX52 and c-Myc expression in three PCa cohorts (Fig. 5c–e). Furthermore, to assess the correlation at the protein level, serial sections of human prostatectomy samples were examined using DDX52 and c-Myc IHC. DDX52 and c-Myc expression was significantly correlated in 86 human PCa specimens (Fig. 5f, g). These results further confirm that DDX52 expression is functionally linked to c-Myc signaling in human PCa.

## Discussion

The role that DEAD/H box RNA helicases play in PCa has been reported by an increasing number of investigators [17–20]. All of these studies have focused on the signaling related to the hormone receptor. Nevertheless, much remains to be explored regarding the functions of DEAD/H box RNA helicases in PCa. This study was undertaken to add on to previous research and discover the role of DDX52 in PCa.

The biological function of DDX52 is poorly understood. Some studies have shown that DDX52 is involved in the process of virus infection [21, 22]. DDX52 has also been reported to contribute to mitosis in spermatogonia and spermatid differentiation [23]. However, there is a lack of reports about DDX52 in cancer-related fields. The present study found an abnormal increase in DDX52 in PCa tissues. As is well-known, different races of PCa patients exhibit huge differences in molecular alterations [24, 25]. However, the patients included in our study were Asian, and combined with results from a previous study on Europeans [9], the data indicate that DDX52 plays a potentially important role in Asian and European PCa patients. It is an emerging challenge to distinguish PCa cases with an aggressive phenotype from those that consist of slow-growing, non-aggressive tumors due to largely indolent PCa. In this regard, DDX52 expression is even higher in metastatic PCa based on other patient cohort studies. Higher DDX52 levels are associated with poorer outcomes in metastatic PCa patients. Moreover, we confirmed that disrupting DDX52 expression attenuated the proliferation of PCa cells in vitro and in vivo. All of these data suggest that DDX52 may have crucial effects on the genesis and progression of advanced PCa. Thus, additional studies are needed to test whether it is useful to stratify PCa cases by DDX52 level for clinical applications.

Previous studies have shown that c-Myc signaling affects various aspects of tumor cell biology, including the cell cycle, stemness, tumorigenesis, invasion, metabolism, tumor-associated immunology, and therapeutic resistance [26–29]. c-Myc is the key oncogene in PCa that changes non-aggressive tumors into aggressive tumors [7, 30]. Nevertheless, the c-Myc signaling-related molecular changes are poorly understood. In this study, the bioinformatics analysis showed that DDX52 expression was positively correlated with c-Myc signaling in PCa tissues, and the result was verified with PCa cells using RNA-seq. Furthermore, we investigated the possible mechanism underlying the effect of DDX52 on c-Myc signaling activity. The c-Myc plays as a key transcription factor to control many genes expression level [28]. The expression of DDX52 might be regulated by c-Myc or c-Myc target genes, such as CDK4, on transcription level. Furthermore, DDX52 is recognized as an RNA helicase, which is possibly contributed to implement the function of c-Myc target genes via regulating mRNA metabolism. We discovered that the regulatory relationship between c-Myc and DDX52 in PCa cells may contribute to the activation of c-Myc signaling by DDX52. Finally, we further validated the positive correlation between expression levels of DDX52 and c-Myc in PCa tissues. Taken together, these findings demonstrate that DDX52 is an important downstream effector of c-Myc that contributes to PCa progression, and c-Myc may rely on DDX52 in PCa cells to carry out its oncogene function. Targeting DDX52 could be a feasible strategy for inhibiting c-Myc-driven PCa tumor growth.

In conclusion, our results reveal the pivotal pro-survival role of DDX52 signaling in PCa cells and verify that there is an abnormal increase in DDX52 in PCa tissues. In addition, our data indicate a regulatory association between DDX52 expression and the activation of c-Myc signaling.

## Supplementary Information

**Additional file 1: Figure S1.** (a) 22RV1 and LNCaP cells were infected with lentiviruses carrying shRNA against DDX52 or shCON, and gene expression was determined using western blotting. (AR-FL: AR full-length, AR-v7: AR variants 7). (b) LNCaP cells were infected with lentiviruses carrying shRNA against DDX52, c-Myc or shCON, and gene expression was determined using western blotting.

## Data Availability

The data and analyzed during the current study are available from the corresponding author on reasonable request.
