# Supplementary figures and images for: DDX52 knockdown inhibits the growth of prostate cancer cells by regulating c-Myc signaling
Source: Cancer Cell Int. 2021 Aug 16;21:430. doi: 10.1186/s12935-021-02128-y (PMC8365980; doi:10.1186/s12935-021-02128-y)

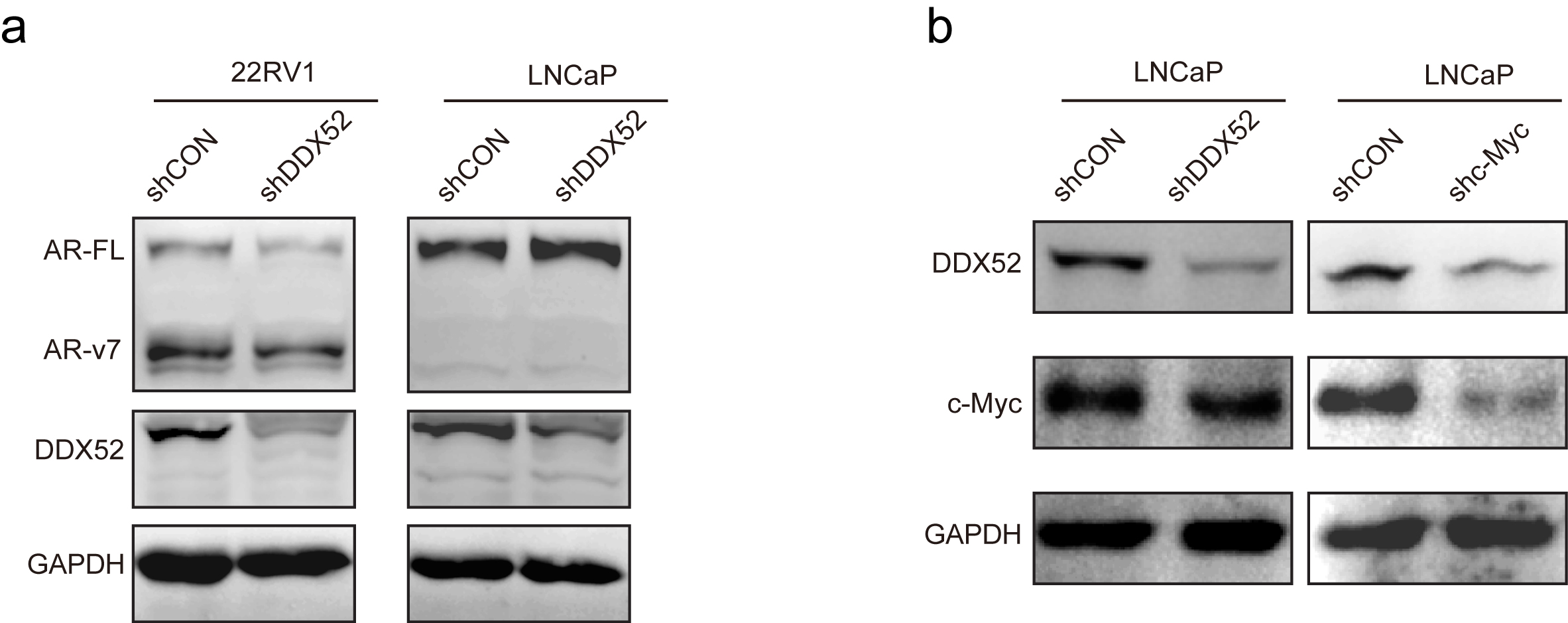

Supplement: Supplementary file 1 — Additional file 1: Figure S1. (a) 22RV1 and LNCaP cells were infected with lentiviruses carrying shRNA against DDX52 or shCON, and gene expression was determined using western blotting. (AR-FL: AR full-length, AR-v7: AR variants 7). (b) LNCaP cells were infected with lentiviruses carrying shRNA against DDX52, c-Myc or shCON, and gene expression was determined using western blotting. [file 12935_2021_2128_MOESM1_ESM.jpg]
